# Reassessment of *Paleotachina* Townsend and *Electrotachina* Townsend and their removal from the Tachinidae (Diptera)

**DOI:** 10.3897/zookeys.361.6448

**Published:** 2013-12-12

**Authors:** James E. O’Hara, Christopher M. Raper, Adrian C. Pont, Daniel Whitmore

**Affiliations:** 1 Canadian National Collection of Insects, Agriculture and Agri-Food Canada, 960 Carling Avenue, Ottawa, Ontario, K1A 0C6, Canada; 2 Angela Marmont Centre for UK Biodiversity, Natural History Museum, Cromwell Road, London SW7 5BD, United Kingdom; 3 Oxford University Museum of Natural History, Parks Road, Oxford OX1 3PW, United Kingdom, and Natural History Museum, Cromwell Road, London SW7 5BD, United Kingdom; 4 Department of Life Sciences, Natural History Museum, Cromwell Road, London SW7 5BD, United Kingdom

**Keywords:** Tachinidae, Muscidae, Sarcophagidae, amber, copal, inclusions

## Abstract

The monotypic genera *Paleotachina* Townsend, 1921 and *Electrotachina* Townsend, 1938 were originally described as fossils in amber but were later discovered to be inclusions in copal. Both taxa were originally assigned to the Tachinidae (Diptera) and this placement has continued to the present day. The holotypes of the two type species, *P. smithii* Townsend and *E. smithii* Townsend, were examined and the following taxonomic and nomenclatural changes are proposed: *Paleotachina* is transferred to the Muscidae and placed in synonymy with *Aethiopomyia* Malloch, 1921, **syn. n.**; *P. smithii* Townsend, type species of *Paleotachina*, is synonymized with *Aethiopomyia gigas* (Stein, 1906), **syn. n.**; *Electrotachina* is transferred to the Sarcophagidae and placed in synonymy with *Dolichotachina* Villeneuve, 1913, **syn. n.**; *E. smithii* Townsend, type species of *Electrotachina*, is recognized as a valid species of *Dolichotachina* **comb. n.** Images of the holotypes of *P. smithii* and *E. smithii* are provided and features that have helped place these copal inclusions in their new combinations are discussed.

## Introduction

For such a large family of Diptera, the Tachinidae have a very meager fossil record. There are about 8500 valid species in the family (O’Hara 2013b), but only ten species in eight genera were listed as fossil Tachinidae by Evenhuis (1994). The oldest of these were presumed to be of Eocene age, thus establishing the Eocene as the minimum age of the Tachinidae.

A preliminary investigation into the authenticity of the presumed oldest tachinid fossils by O’Hara (2013a) called into question the family identifications of the three taxa involved: *Vinculomusca vinculata* (Scudder), *Paleotachina smithii* Townsend, and *Electrotachina smithii* Townsend. The first was described from “part of emptied skins” of dipteran larvae preserved in rock and originating from Chagrin Valley, Colorado (Scudder 1877). The species was originally described in *Musca* Linnaeus, but Townsend (1938) erected the new genus *Vinculomusca* for it and declared it of “apparently exoristid or tachinid stock” (i.e., Tachinidae). As noted by O’Hara (2013a), there is insufficient evidence to place the fossilized larval remains to family and assignment to the Tachinidae—the larvae of which are arthropod endoparasitoids—is especially unmerited.

Treated in this paper are the monotypic genera *Paleotachina* and *Electrotachina*. Both were described by Townsend (1921, 1938) based on figures in Smith (1868). As explained below, they were until relatively recently thought to be Baltic amber fossils but are now known to be much younger specimens preserved in East African copal. Our examination of the holotypes of the two species involved, *Paleotachina smithii* and *Electrotachina smithii*, has confirmed O’Hara’s (2013a) suspicion that neither belongs to the Tachinidae. Their identities are discussed and the appropriate taxonomic and nomenclatural changes are proposed. Images of the type specimens are provided.

## Materials and methods

**Figures 1–6.**
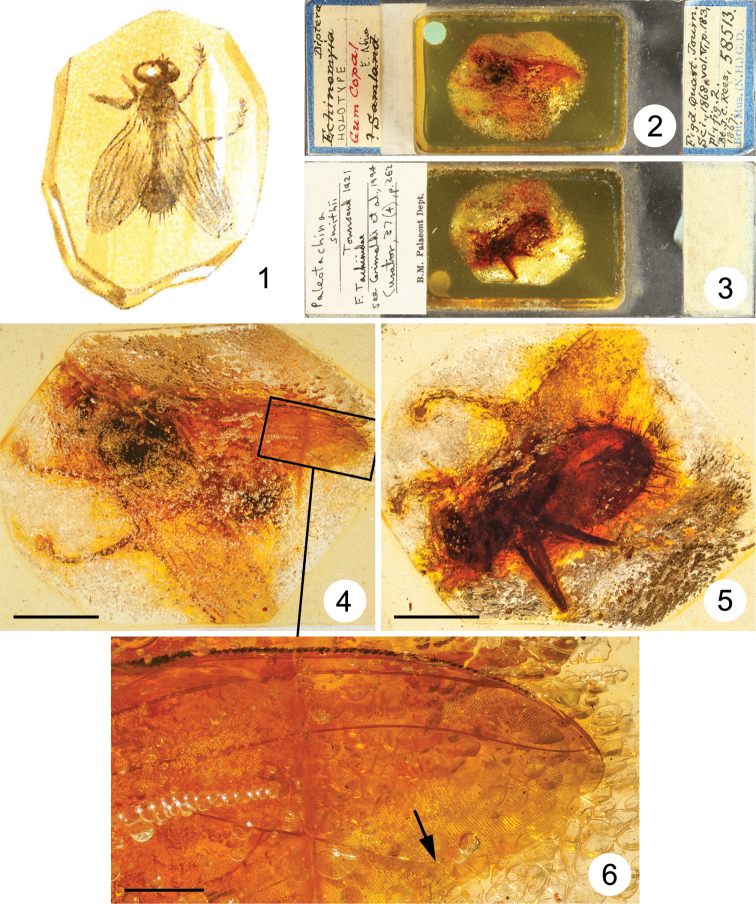
*Paleotachina smithii* Townsend, 1921 (junior synonym of *Aethiopomyia gigas* (Stein, 1906), syn. n.), Muscidae **1** reproduction of illustration in Smith (1868, fig. 2) showing inclusion originally identified as “*Echinomyia*” sp. (i.e., *Echinomya* Latreille, 1805, junior synonym of *Tachina* Meigen, 1803, Tachinidae) **2–6** holotype male **2–3** entire slide **2** dorsal view **3** ventral view **4–6** inclusion **4** dorsal view (scale bar = 5.0 mm) **5** ventral view (scale bar = 5.0 mm) **6** enlarged portion of wing circumscribed in Fig. 4 (arrow indicates bend of vein M) (scale bar = 1.0 mm).

**Figures 7–13.**
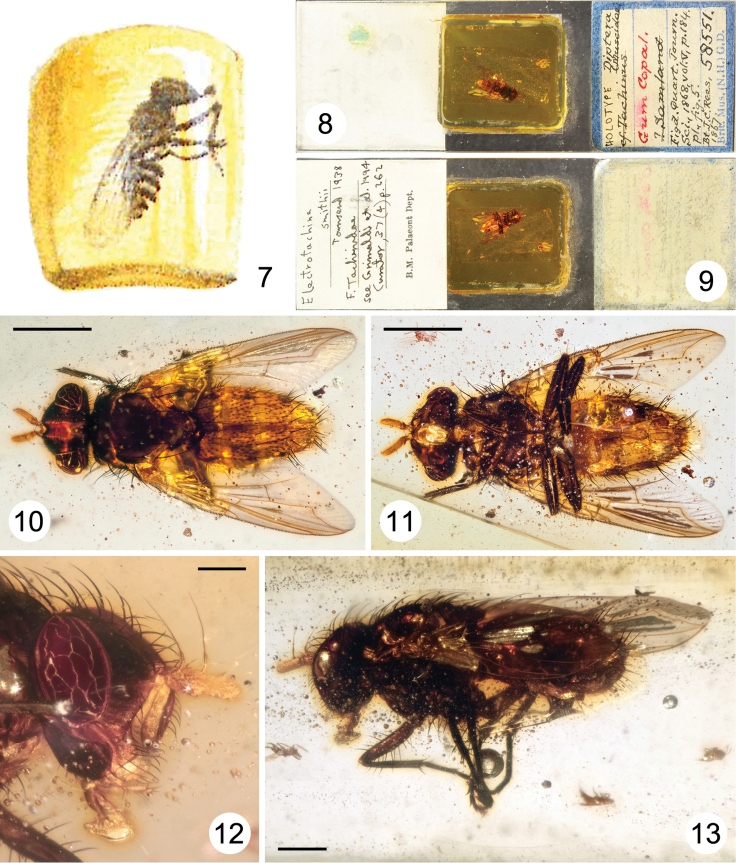
*Electrotachina smithii* Townsend, 1938 (now *Dolichotachina smithii* (Townsend, 1938), comb. n.), Sarcophagidae **7** reproduction of illustration in Smith (1868, fig. 5) showing inclusion originally identified as a new genus near “*Tachinus*” (i.e., *Tachina* Meigen, 1803, Tachinidae) **8–13** holotype female **8–9** entire slide **8** dorsal view **9** ventral view **10–13** inclusion **10** dorsal view (scale bar = 2.0 mm) **11** ventral view (scale bar = 2.0 mm) **12** head, right lateral view (scale bar = 0.5 mm) **13** body, left lateral view (scale bar = 1.0 mm).

The holotypes of *Paleotachina smithii* and *Electrotachina smithii* are deposited in the Natural History Museum, London, United Kingdom (NHM). One of us (AP) studied the holotype of *Paleotachina smithii* and another (DW) studied the holotype of *Electrotachina smithii*, thus allowing these inclusions to be placed with some confidence to the species or genus level within the Muscidae and Sarcophagidae, respectively. Each specimen is preserved within a small piece of copal, which is in turn embedded in Canada balsam within a square open-topped glass case glued to a slide. The glass case containing *Electrotachina smithii* was covered with a cover slip following the recent restoration of the Canada balsam surface, which was scratched. Images for Figs 2–3 and 8–9 were taken with a Canon EOS 550D camera fitted with a Canon MP-E 65 mm lens; images for Figs 4–6 and 10–11 were taken with a Canon EOS 5D Mark II camera fitted with a Canon MP-E 65 mm lens; images for Figs 12–13 were taken with a Canon EOS 650D camera fitted with a 0.63x adaptor mounted on a Leica MZ125 stereomicroscope. Images for Figs 4–6 and 10–13 were stacked using Helicon Focus (version 5.3) software. Figures 1 and 7 were scanned from a plate in Smith (1868) and their low resolution is a reflection of the poor quality of the plate in the original publication.

### Age of fly inclusions depicted in Smith (1868)

The paper by Zaddach (1868) was a detailed account of the origins of the amber deposits of “Samland”, an area known today as the Samland Peninsula in Kaliningrad Oblast, Russia. This area is the richest source for Baltic amber, which is mined locally or erodes out of deposits under the Baltic Sea and washes ashore. Zaddach (1868) referred to the age of the deposits as “Eocene or Lower Oligocene”. Modern dating methods have established an Eocene origin for Baltic amber with an age of about 44 Ma (Engel 2001).

The editors of the *Quarterly Journal of Science* followed Zaddach’s (1868) paper with a plate meant to “convey some idea of the organic remains usually found in this fossil resin” (p. 183) and a list of works on amber and inclusions. The editors assumed responsibility for both the plate and list of works, but also noted (p. 183): “The specimens figured in that plate belong to the National Collection in the British Museum; and for the facts relating to the Insects embodied in the annexed explanation of it, we are indebted to the kind and able assistance of Mr. Frederick Smith, of the Entomological Department of that Museum”. For the purposes of bibliographic reference, both the plate and the explanation of it are cited here as Smith (1868).

Neither Smith (1868) nor the editors of the *Quarterly Journal of Science* gave the provenance of the “amber” pieces depicted in the plate but subsequent authors assumed the pieces originated from Baltic deposits and were authentic amber of the age suggested by Zaddach (1868). This is evident in the descriptions of *Paleotachina* and *Electrotachina* by Townsend (1921, 1938, 1942) and in later works citing these taxa, for example Spahr (1985), Evenhuis (1994), Lehmann (2003), and O’Hara (2013a). However, in a semi-popular paper on *Forgeries of Fossils in “Amber”* overlooked by Lehmann (2003) and O’Hara (2013a), Grimaldi et al. (1994) discussed Smith’s (1868) inclusions and changed both their age and origin. The ten pieces containing arthropods had been purchased by the British Museum (Natural History) (now NHM) in 1867 and were thought at the time to have originated from Baltic deposits in the vicinity of “Stettin” (present-day Szczecin in Poland) (Grimaldi et al. 1994). In truth, the pieces are copal from East Africa (Grimaldi op. cit.). Further details about the age of the copal or the location where it was found in East Africa are lacking.

## Systematics

### Aethiopomyia

Malloch, 1921

http://species-id.net/wiki/Aethiopomyia

(Muscidae)

Aethiopomyia Malloch, 1921: 426. Type species: *Spilogaster gigas* Stein, 1906 (as “*Mydaea gigas*, Stein”), by original designation.
Paleotachina Townsend, 1921: 134. Type species: *Paleotachina smithii* Townsend, 1921 (= *Spilogaster gigas* Stein, 1906, syn. n.), by monotypy. Syn. n.
Palaeotachina . Incorrect subsequent spelling of *Paleotachina* Townsend, 1921 (Evenhuis 1994: 467, Lehmann 2003: 116, O’Hara 2013a: 11, 12).

#### Remarks.

The genus-group names *Aethiopomyia* and *Paleotachina* were both made available in 1921. The paper by Malloch (1921) was published on May 1 (Evenhuis 2003) and the paper by Townsend on October 3 (Evenhuis 1994), thus giving date priority to *Aethiopomyia*.

### Aethiopomyia gigas

(Stein, 1906)

http://species-id.net/wiki/Aethiopomyia_gigas

Figs 1–6

Spilogaster gigas Stein, 1906: 37. Syntypes, 1 male and 2 females (Museum für Naturkunde der Humboldt-Universität zu Berlin, Berlin; seen by Pont 2013: 77). Type locality: Cameroon, Barombi.
Paleotachina smithii Townsend, 1921: 134. Holotype male, in copal (NHM, No. 58513). Type locality: East Africa (Grimaldi et al. 1994). Syn. n.

#### Remarks.

Smith (1868: 183), in his explanation of a plate of “amber” inclusions, wrote the following caption for the specimen that later became the holotype of *Paleotachina smithii*: “Fig. 2.—A Dipterous Insect belonging to the European genus *Echinomyia*. Enlarged one-half”. Based on this caption and the figure itself, Townsend (1921: 134) wrote the following for his new genus and species: “*Paleotachina* gen. nov. *smithii* sp. nov. (fossil).—Proposed for *Echinomyia* sp. Smith (1868), Qu. Jn. Sc. V, 183, f. 2. From the Lower Oligocene of Baltic amber. The description indicates one of the Larvaevorini or allied tribes”.

The “Larvaevorini” of Townsend (1921) later became known as the Tachinini when *Larvaevora* Meigen, 1800 was suppressed by ICZN (1963). Although the species *Paleotachina smithii* was not described by Townsend (or by Smith, despite Townsend’s statement to the contrary), the species-group name was made available by bibliographic reference to fig. 2 in Smith (1868) (Article 12.2.1 of ICZN 1999). Townsend (1942: 17) later provided a brief description of the genus, presumably from fig. 2 in Smith (1868), and referred to the genus as “evidently tachinid”.

A considerable amount of artistic liberty was taken in the depiction of NHM specimen #58513 (holotype of *Paleotachina smithii*) in fig. 2 in Smith (1868), which was also shown as a mirror image of the original specimen; cf. Figs 1, 4.

The holotype of *Paleotachina smithii* is a large fly in the family Muscidae, with a body length of about 14 mm and a wing length of about 14 mm. It is well preserved, but large parts of it are obscured by masses of small air bubbles (see Figs 2–5). The conformation of the abdominal tip suggests that it is a male, but nothing can be seen of the head and associated features. Because of its size, coloration and habitus, the presence of very long stout setae on abdominal tergites 4 and 5, and a vein M that is weakly curved forward towards vein R_4+5_ in its apical part (Fig. 6), leaving a wide open cell r_4+5_, the species can be readily assigned to either *Aethiopomyia* Malloch or *Alluaudinella* Giglio-Tos, two genera confined to the Afrotropical Region. It is possible to see several small setulae on the node at the base of vein R_4+5_, and such setulae are present in *Aethiopomyia* but not in *Alluaudinella*. Other characters used to differentiate these genera (proepisternal depression setulose or bare, katatergite with fine setulae or bare) cannot be seen in the holotype.

The scutum, scutellum and at least abdominal tergites 4 and 5 are black; the remainder of the body (the head excepted) is yellow. The femora and tibiae are yellow, and the tarsi black. This coloration is most similar to that of *Aethiopomyia gigas* (Stein), described from Cameroon and widespread though never common across western, eastern and southern Africa. *Paleotachina smithii* Townsend, 1921 is accordingly synonymized with *Aethiopomyia gigas* (Stein, 1906), syn. n.

### Dolichotachina

Villeneuve, 1913

(Sarcophagidae)

Dolichotachina Villeneuve, 1913: 112. Type species: *Tachina marginella* Wiedemann, 1830, by monotypy.
Electrotachina Townsend, 1938: 166. Type species: *Electrotachina smithii* Townsend, 1938, by original designation. Syn. n.

### Dolichotachina smithii

(Townsend, 1938) comb. n.

http://species-id.net/wiki/Dolichotachina_smithii

Figs 7–13

Electrotachina smithii Townsend, 1938: 166. Holotype female, in copal (NHM, No. 58551). Type locality: East Africa (Grimaldi et al. 1994).

#### Remarks.

Townsend (1938: 166) began his description of *Electrotachina* with: “Genotype, *Electrotachina smithii* sp. nov. For new genus Muscidae aff. *Tachina* sp. F. Smith, Quart. Jn. Sc., V, 184, pl. 18, fig. 5 (1868). Fly Lower Oligocene of Baltic amber”. A brief description followed, ending with the statement “probably exoristid or tachinid stock”. There is no indication that Townsend examined the specimen and his description is consistent with the drawing of a fly in fig. 5 in Smith (1868). As with *Paleotachina*, Townsend (1942: 12) later provided a brief description of the genus, presumably from fig. 5 in Smith (1868), and referred to the genus as “almost certainly exoristid stock”.

As with *Paleotachina smithii*, certain liberties were taken in the depiction of NHM specimen #58551 (holotype of *Electrotachina smithii*) in fig. 5 in Smith (1868), and it may also have been shown as a mirror image of the original specimen; cf. Figs 7, 13.

*Electrotachina smithii* belongs to the family Sarcophagidae, subfamily Miltogramminae. The holotype female has a body length of about 7 mm and is preserved in a small block of copal (approx. 15 × 10 × 7 mm) together with two other adult dipterans: a small female Agromyzidae and a Cecidomyiidae. The specimen is in very good condition except for the lack of its right fore tarsus. Antennae, wings and chaetotaxy are all in excellent condition. The specimen can be confidently assigned to the genus *Dolichotachina* based on the following combination of external character states: arista short pubescent, thickened on approximately basal 1/5; eye bare; parafacial with an uneven row of setae anteriorly; proepisternum bare; katepisternum with two, widely separated, setae; mid tibia with one anterodorsal seta; wing cell r_4+5_ open at wing margin.

In addition to the above features, *Dolichotachina smithii* is characterized by an elongated postpedicel (about 3 times length of pedicel), relatively short vibrissa, and short proboscis (about twice as long as wide).

*Dolichotachina* is a mainly Afrotropical genus with 12 species previously known from this region (Pape 1996). Material recently collected in Burundi and Namibia has demonstrated that the Afrotropical diversity of *Dolichotachina* is probably greatly underestimated (Whitmore & Pape, unpublished). The condition of the holotype and the difficulty of identifying *Dolichotachina* females have not allowed us to verify whether this specimen is conspecific with any of the other described species. Lacking any strong indication to the contrary, we consider *Dolichotachina smithii* to be a valid, probably extant, species from East Africa.

### Age of the Tachinidae

Fossils are the most reliable indicators of the minimum age of the lineage to which they belong, but they provide false information if they are incorrectly identified or dated. As explained above, the minimum age of the Tachinidae is no longer the Eocene based on fossil evidence. Instead, the oldest fossils date the family to the Oligocene (Evenhuis 1994), assuming those fossils are accurately identified and dated. Von Tschirnhaus and Hoffeins (2009) reported on a dipteran in Baltic amber that might belong to the Tachinidae but it is in such poor condition that even tachinid specialist H.-P. Tschorsnig (Stuttgart) could not be sure of its placement to family.

The merging of phylogenetic data with data from fossils of known age and identity to create chronograms is becoming more common in evolutionary studies. The results are generally speculative but provide an estimated evolutionary timeline that can be further refined and tested by future research. Two recent studies on the Diptera have suggested different ages for the origin of the Tachinidae. One, a large study by Wiegmann et al. (2011), estimated the origin of the Tachinidae at about 30 million years ago (mya) (i.e., mid Oligocene). The other, by Zhao et al. (2013) and based on fewer data, suggested the Tachinidae originated about 48 mya (i.e., mid Eocene). This latter estimate was tempered by a broad confidence interval. Neither of these estimates is inconsistent with the re-assessed fossil record of Tachinidae, which does not contribute towards an understanding of the age of the family beyond that of the minimum age.

## Supplementary Material

XML Treatment for Aethiopomyia

XML Treatment for Aethiopomyia gigas

XML Treatment for Dolichotachina

XML Treatment for Dolichotachina smithii
